# Left atrial conduit function: A short review

**DOI:** 10.14814/phy2.15053

**Published:** 2021-10-04

**Authors:** Paolo N. Marino

**Affiliations:** ^1^ School of Medicine Università del Piemonte Orientale Novara Italy; ^2^ Istituto Iperbarico Villafranca (Verona) Italy

## Abstract

Three‐dimensional echocardiography can elucidate the phasic functions of the left atrium if a simultaneous acquisition of a pyramidal full‐volume dataset, as gathered from the apical window and containing the entire left atrial and left ventricular cardiac sections, is obtained. Hence, conduit can be quantified as the integral of net, diastolic, instantaneous difference between synchronized atrial and ventricular volume curves, beginning at minimum ventricular cavity volume and ending just before atrial contraction. Increased conduit can reflect increased downstream suction, as conduit would track the apex‐to‐base intracavitary pressure gradient existing, in early diastole, within the single chamber formed by the atrium and the ventricle, when the mitral valve is open. Such a gradient increases in response to adrenergic stimulation or during exercise and mediates an increment in passive flow during early diastole, with the ventricle being filled from the atrial reservoir and, simultaneously, from blood drawn from the pulmonary veins. In this context conduit, and even more conduit flow rate, expressed in ml/sec, can be viewed as an indirect marker of left ventricular relaxation.

It is well known, however, that a large amount of conduit (in relative terms) is also supposed to contribute to LV stroke volume in conditions of increased resistance to LV filling, when diastolic function significantly worsens. Stiffening of the atrio‐ventricular complex implies increments in LA pressure more pronounced in late systole, causing markedly elevated “v” waves, independently of the presence of mitral insufficiency. The combination of increased atrio‐ventricular stiffness and conduit flow is associated with an elevation of the right ventricular pulsatile relative to resistive load that negatively impacts on exercise capacity and survival in these patients.

Atrial conduit is an “intriguing” parameter that conveys a noninvasive picture of the complex atrioventricular coupling condition in diastole and its backward effects on the right side of the heart and the pulmonary circulation. Given the easiness associated with its correctly performed quantification in the imaging laboratory, I am sure that conduit will survive the competitive access to the list of valuable parameters capable of deciphering, although not necessarily simplifying, the complex diastolic scenario in health and disease.

## Graphical abstract

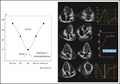

The left atrial (LA) cavity, which connects the pulmonary circulation with the corresponding ventricle, is characterized by phasic activity which makes the description of the atrium as of a passive player in the complex scenario of the cardiac cycle unrealistic (Maccio' & Marino, 2008). This cavity, in fact, is strictly related to left ventricular (LV) function throughout the entire cardiac cycle (Braunwald & Frahm, 1961). After QRS complex at ECG takes place, the cardiac base is forced to descend, due to the longitudinal component of fiber shortening forces associated with LV contraction, contributing to LA filling from the pulmonary veins (Castello et al., 1991). Such *reservoir* function can be quantified as the difference between maximum and minimum volume of the cavity. During late diastole, the atrium also actively contributes to ventricular filling. Such *pump* function can be defined as the blood volume pushed into LV during atrial systole, plus the volume of the backward flow into the pulmonary veins. Finally, during early and mid‐diastole, the atrium passively contributes to LV filling (*conduit* function). During this phase, the cavity is directly exposed to the LV pressure through the open mitral valve and conduit flow is obviously and strongly influenced by the left heart diastolic properties and the gradient relative to the pulmonary venous compartment (Kono et al., 1992), with conduit defined as [LV filling volume − (LA reservoir + pump volume)]. Obviously, in absolute terms, the total amount of blood handled by the atrium acting as a reservoir, pump, and conduit has to equal the ventricular filling volume (Maccio' & Marino, 2008).

Stiffening of the atrioventricular complex implies increments in LA pressure more pronounced in late systole, causing markedly elevated “v” waves (Urey et al., 2017), a phenomenon not necessarily mediated by the presence of mitral insufficiency. More than 15 years ago, we studied a group of 15 patients instrumented during open heart surgery with open pericardium and dichotomized the group according to the median value of invasively assessed LA stiffness (≤ or >0.33 mmHg/ml; Marino et al., 2004). We could demonstrate that a higher stiffness estimate (0.75 ± 0.43 mmHg/ml) was associated with an increased “v” wave and a subsequent larger “y” atrial pressure descent (−9.3 ± 5.6 mmHg) as compared with the group with a lower stiffness value (0.19 ± 0.10 mmHg/ml), in whom the “y” pressure descent was much less (−1.2 ± 0.6 mmHg, *p* < 0.001; Marino et al., 2004).

Such higher pressure pulsatility inside the atrial chamber, mediated by increased cavity stiffness, was also associated with a larger cumulative pulmonary vein flow (51 ± 30 ml/m^2^ vs. 27 ± 9 ml/m^2^, *p* = 0.04) during mitral valve E‐wave acceleration (table 2 of Marino et al., 2004). Thus, LA cavity properties govern the pulsatile profile existing inside the chamber, while modulating conduit flow, with both factors, pulsatility and conduit, being inversely related to atrial compliance.

Such considerations anticipate the consequences of a poorly performing ventricle on both LA reservoir, due to an attenuated cardiac base descent contributing to increased LA pulsatility, and conduit, secondary to an impaired downstream suction, counteracted by compensatory E‐wave PV flow.

## 1.1 Atrial stiffness modulation of exercise capacity in heart failure patients

It is well known that in heart failure (HF) patients prognosis is strongly associated with the performance of the right ventricular (RV) cavity and its loading conditions (Vonk Noordegraaf et al., 2019). Less known, however is that RV afterload is determined by a pulsatile component, represented by pulmonary artery compliance (PAC), in addition to the steady component indexed by pulmonary vascular resistances (PVR; Tedford, 2013). These two parameters, PVR and PAC, are closely related by an inverse relationship designated by the time‐constant of the pulmonary circulation (RC‐time; Lankhaar et al., 2006).

This relationship, between the pulsatile and steady components, is believed to be rather constant, although it has been shown that such constancy can be impacted by LA pressure, when pressure (and stiffness) may be abnormally elevated like in HF patients. Tedford et al. (2012), in fact, have demonstrated that elevation of pulmonary wedge pressure shifts the PAC–PVR hyperbolic curve leftward and downward, foreshortening that LA pressure elevation augments RV pulsatile relative to resistive load. Such a shift in pressure components, from a steady to a pulsatile prevalent contribution, has important functional and prognostic significance. In HF patients, elevated LA pressure is, in fact, associated with a shift of the PAC–PVR relationship, such that the pulsatile component becomes augmented and contributes to RV afterload, despite a relatively “normal” steady component (Najjar et al., 2021). This may limit RV output, and negatively impact exercise capacity and survival in these patients (Zanaboni et al., 2021).

## 1.2 Conduit and left heart diastolic dysfunction

**FIGURE 1:**
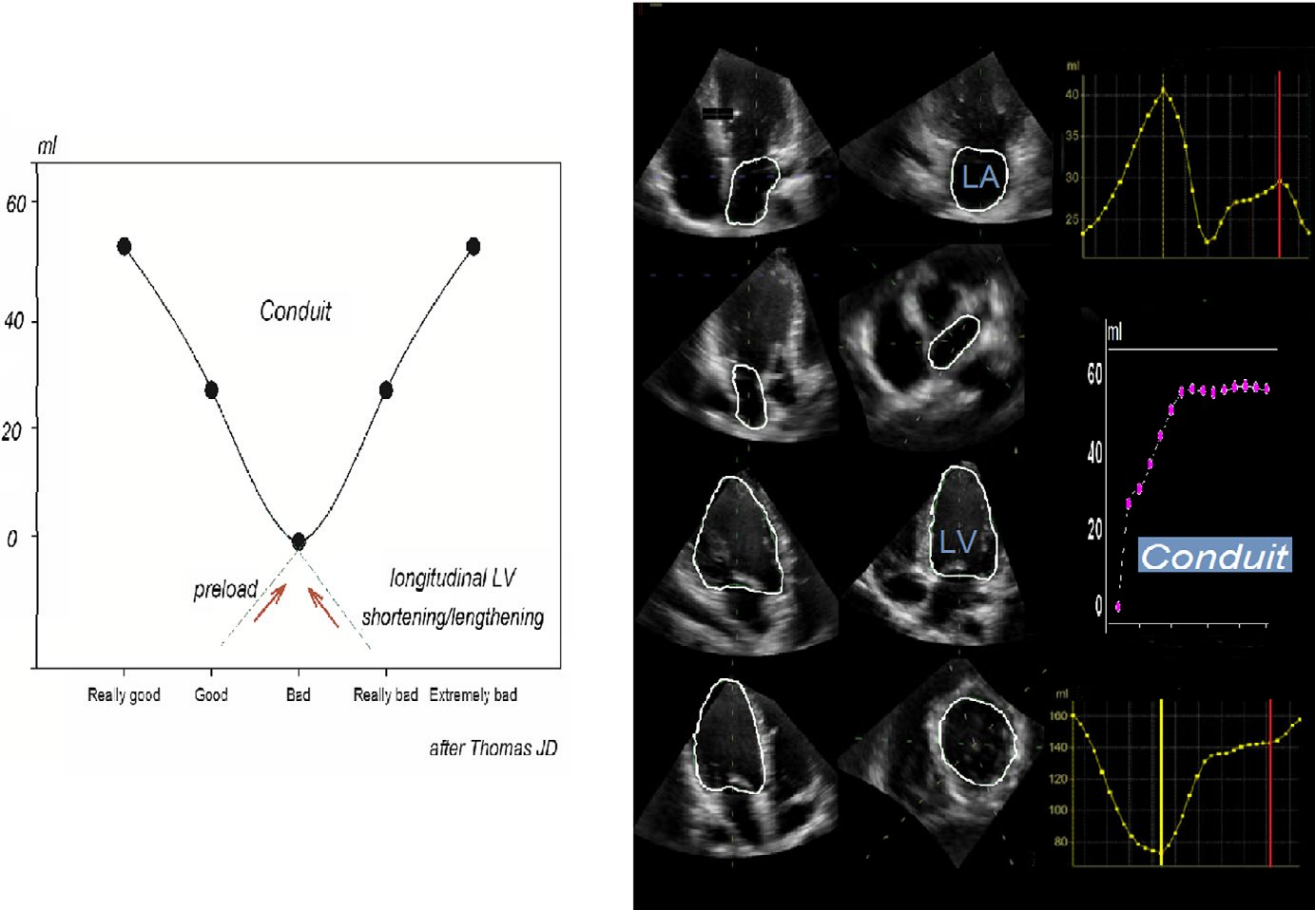
Left: Behavior of conduit according to changes in preload and/or changes in ventricular longitudinal shortening/lengthening (see text for details). Right: 3D images of the left atrial (LA) and left ventricular (LV) cavity (with endocardial border delineated), with corresponding continuous LA (upper right panel) and LV (lower right panel) volume curves. Conduit flow volume curve (middle right panel) is computed as [LV volume (time) − LV minimum volume] − [LA maximum volume − LA volume (time)], from minimum LV volume (yellow vertical continuous line) to ECG P wave (red vertical line). Conduit starts from 0 ml, at minimum LV cavity volume, and then it reaches a plateau in mid‐diastole, ending before LA cavity contractio

It is well known that a large amount of conduit is supposed to contribute to LV stroke volume in conditions of increased resistance to LV filling (and this would explain why conduit contribution to LV stroke volume would increase with worsening diastolic dysfunction; Marino et al., 2019). Less obvious, however, is that such an augmented conduit contribution may also be found in a totally opposite scenario, with conduit progressively contributing to filling volume during intensive exercise in competitive athletes (Wright et al., 2015). This is compatible with a U‐shaped behavior for this parameter across the spectrum (Figure 1, left), from a markedly diseased condition, like end‐stage HF, to a “supranormal” situation, like the one depicted in athletes, similarly to what happens for other functional imaging parameters (Al‐Mashat et al., 2020). In the “supranormal” scenario, in fact, conduit would track the apex‐to‐base intracavitary pressure gradient existing, in early diastole, within the single chamber formed by the atrium and the ventricle, when the mitral valve is open (Courtois et al., 1988). Such a gradient increases with the augmentation of the LV longitudinal contraction and subsequent lengthening that develops in response to adrenergic stimulation or during exercise (Levy et al., 1993; Nonogi et al., 1988; Ohara et al., 2012) and mediates an increment in passive flow during early diastole, with the ventricle being filled from the atrial reservoir and, simultaneously, from blood drawn from the pulmonary veins. In this context conduit, and even more conduit flow rate, expressed in ml/s, can be viewed as an indirect marker of LV relaxation (Bhatt et al., 2020; Marino et al., 2021).

According to the above reasoning increments in conduit flow may reflect increased suction, acting on the LV side, or exaggerated filling and pulsatile pressure, acting on the LA side (Figure 1, left), along with increased recoil of the pulmonary vasculature. This makes interpretation of this type of parameter not easy, but nonetheless informative of the complex scenario existing in case of diastolic dysfunction/hyperfunction.

## 1.3 How conduit can be quantified?

It is challenging to quantitatively separate the rapid filling volume of blood entering the LV in early diastole into the conduit versus reservoir components. More than 2 decades ago, in order to quantify conduit, we proposed an approach that integrated the information derived from the Doppler mitral flow velocity profile with that of one single pulmonary vein (taken as a representative of all the four veins; Prioli et al., 1998). In that study we demonstrated the existence of an inverse linear relationship between the conduit contribution to LV filling and a “classical” diastolic descriptor, that is, the Doppler E‐wave deceleration time. The approach we used, however, involved several assumptions and did not allow us to delve further into the potential role of atrial conduit function itself as an index of the diastolic condition.

More recently, we adopted 3D echocardiography, gathering simultaneous acquisitions of a pyramidal full‐volume dataset from the apical window and containing the entire LA and LV cardiac sections (Nappo et al., 2016). After optimal image alignment and manual contouring of the ventricular and atrial endocardium at the beginning of the cardiac cycle, software can produce, automatically, LV and LA volume curves along the entire subsequent beat. From these curves, that can be manually edited if necessary, conduit can be computed (provided mitral and/or aortic insufficiency is trivial) as the integral of the net, diastolic instantaneous difference between synchronized atrial and ventricular volume curves, beginning at minimum LV cavity volume and ending just before atrial contraction, synchronously with P wave on the electrocardiographic trace (Figure 1, right; Bowman & Kovacs, 2004; Nappo et al., 2016). Obviously, conduit flow rate can also be obtained, at baseline or during exercise, referencing the cumulative conduit flow to the above defined time interval.

Whatever the way conduit is nominally expressed, it must be emphasized that the amount of blood entering LV from the atrium during diastole *cannot* be faithfully reflected by the atrial volume curve alone. In the phase of passive atrial emptying and atrial diastasis pulmonary veins drain blood from the lungs into the ventricle. Further, not to forget is that, during atrial contraction, some blood does flow back into the pulmonary veins, although it is known that sleeve contraction should normally limit this retrograde flow to a small amount (Thiagalingam et al., 2008). Thus, it is only the simultaneous availability of both (atrial and ventricular) cavity volume curves that guarantees a precise definition of the atrial conduit contribution to LV filling (Marino, 2010).

It has been anticipated that simultaneous volumetric measurements of LV and LA cavity is not possible with 2D images because the LV major axis differs from LA major axis (Lang et al., 2015). Since in 3D the entire heart is scanned, the volumes of both the LV and LA can be carefully measured in one acquisition (Nappo et al., 2016). This would be particularly important in patients in atrial fibrillation, when the assessment of the atrioventricular volumetric interaction during diastole can be soundly investigated by 3D echocardiography (Otani et al., 2016). In this line of reasoning, we did recently show that conduit quantitation precardioversion is able to predict early arrhythmia recurrence in persistent atrial fibrillation patients (Giubertoni et al., 2019). These findings support the concept that conduit quantitation is valuable as it can reflect diastolic LA pathology that cannot necessarily be explained by ventricular pathology only and, thus, it could be also proposed as a clinically effective tool for exploring the link between AF and diastolic dysfunction, in excess of ventricular derangement (Degiovanni et al., 2018; Roeder et al., 2017).

## 1.4 Conclusion

Conduit is an “intriguing” parameter that conveys a noninvasive picture of the complex atrioventricular coupling condition in diastole and its backward effects on the right side of the heart and the pulmonary circulation. Given the easiness associated with its correctly performed quantification in the imaging laboratory, I am sure that conduit will survive the competitive access to the list of valuable parameters capable of deciphering, although not necessarily simplifying, the complex diastolic scenario in health and disease (Nishimura & Tajik, 1997).

## CONFLICT OF INTEREST

The author confirms that he has no conflict of interest to declare in relation to the submitted paper.

## AUTHOR CONTRIBUTION

Paolo N. Marino conceived, drafted, and finalized the manuscript to be published.
